# Advancements in Percutaneous Coronary Intervention Techniques: A Comprehensive Literature Review of Mixed Studies and Practice Guidelines

**DOI:** 10.7759/cureus.41311

**Published:** 2023-07-03

**Authors:** Muhammad Abubakar, Izzah Javed, Hafiz Fahad Rasool, Saud Raza, Deepak Basavaraju, Rai Muhammad Abdullah, Faizan Ahmed, Siffat S Salim, Muhammad Ahmad Faraz, Khawaja Mushammar Hassan, Mohsin Hajjaj

**Affiliations:** 1 Department of Internal Medicine, Ameer-ud-Din Medical College/Lahore General Hospital, Lahore, PAK; 2 Department of Internal Medicine, Siddique Sadiq Memorial Trust Hospital, Gujranwala, PAK; 3 Department of Public Health, School of Public Health, Nanjing Medical University, Nanjing, CHN; 4 Department of Internal Medicine, Mysore Medical College and Research Institute, Mysore, IND; 5 Department of Anesthesia & ICU, Punjab Social Security Hospital, Lahore, PAK; 6 Department of Surgery, Holy Family Red Crescent Medical College Hospital, Dhaka, BGD; 7 Department of Forensic Medicine, Post Graduate Medical Institute, Lahore General Hospital, Lahore, PAK; 8 Department of Internal Medicine, Jinnah Hospital, Lahore, PAK

**Keywords:** quality indicators, practice guidelines, percutaneous coronary intervention, drug-eluting stents, coronary artery disease, bioresorbable vascular scaffolds, advancements

## Abstract

Percutaneous coronary intervention (PCI) is a widely used therapy for coronary artery disease (CAD), but it carries risks and complications. Adhering to evidence-based practice guidelines is crucial for optimal outcomes. This review compares the recommendations of the 2021 American College of Cardiology/American Heart Association/Society for Cardiovascular Angiography and Interventions (ACC/AHA/SCAI) and 2018 European Society of Cardiology (ESC) guidelines for coronary artery revascularization and discusses emerging trends and novel devices in PCI. A comprehensive literature review of mixed studies, clinical trials, and guidelines was conducted. Intravascular imaging, including intravascular ultrasound and optical coherence tomography, for stent optimization, is also recommended when feasible. However, differences reflecting variations in evidence quality interpretation and applicability were identified. Furthermore, novel devices and technologies with the potential for improving outcomes were highlighted, but their safety and efficacy compared to standard-of-care techniques require further evaluation through extensive randomized trials. Clinicians should stay updated on advancements and personalize treatment decisions based on individual patient factors. Future research should address evidence gaps and barriers to adopting innovative devices and techniques. This review provides recommendations for clinical practice, emphasizing the need to remain current with the evolving landscape of PCI to optimize patient outcomes. The discoveries provide valuable counsel for the deliberation of clinical interventions and prospective inquiries within the realm of interventional cardiology. Overall, the review underscores the importance of evidence-based practice and ongoing advancements in PCI for CAD management.

## Introduction and background

Percutaneous coronary intervention (PCI) is an invasive yet non-operative procedure designed to alleviate the constriction or blockage of the coronary artery, thus enhancing blood flow to the heart muscle. PCI is essential in managing a primary cause of mortality worldwide, coronary artery disease (CAD). CAD is characterized by plaque accumulation within the coronary arteries, reducing their diameter and limiting oxygen delivery to the heart. The reduced oxygen delivery can result in angina (chest pain), shortness of breath, or even a full-blown myocardial infarction (MI). PCI can help restore normal blood flow and relieve symptoms of CAD [1].

The history of PCI can be traced back to 1977 when Andreas Gruentzig first introduced balloon angioplasty. This technique requires placing a balloon catheter into an obstructed artery and expanding it to push the plaque against the artery walls, thus widening the artery and improving blood flow. Over time, PCI has evolved to include more sophisticated techniques, including drug-eluting stents (DES) and intracoronary stenting [1,2]. The choice of a stent for a patient, whether it is drug-coated or not, depends on their risk factors and preferences.

PCI has become one of the most common treatments for CAD worldwide, with more than two million procedures performed annually. However, PCI has challenges and limitations. Some of these include restenosis (re-narrowing of the artery), stent thrombosis (clotting inside the stent), bleeding complications, radiation exposure, and cost-effectiveness [1-3]. Therefore, ongoing research and innovation are needed to improve PCI outcomes and safety.

Advancements in PCI techniques have significantly improved patient outcomes, with reduced complications and better long-term results. Some of the latest advancements in PCI techniques include bioresorbable vascular scaffolds (BVS), which gradually dissolve after the artery has healed, and the adoption of new imaging technologies, including intravascular ultrasound (IVUS) and optical coherence tomography (OCT), which reinforce precise decision-making and treatment planning [4]. Additionally, robotics and telemedicine are making PCI procedures more accessible to patients in remote areas [5].

PCI has undergone remarkable developments in the past decades, with new devices, techniques, and strategies improving its efficacy and safety. Ongoing research in PCI aims to improve patient outcomes further and make PCI procedures more accessible to patients worldwide. This article will provide a comprehensive literature review of various studies, clinical trials, and practice guidelines on advancements in PCI techniques, emphasizing new devices, imaging modalities, pharmacological agents, procedural strategies, patient selection criteria, and quality indicators for PCI. This comprehensive assessment will further consolidate the existing knowledge foundation and exemplary protocols concerning PCI in individuals afflicted with CAD. Additionally, it will impart valuable perspectives into the forthcoming trajectory of PCI for CAD patients.

## Review

PCI has become the standard for treating CAD. It involves using devices and pharmacological agents to restore blood flow in blocked or narrowed coronary arteries. Recent advancements in PCI techniques have focused on improving patient outcomes, reducing complications, and increasing procedural success rates. These advancements include 1) new devices, 2) imaging modalities, 3) pharmacological agents, 4) procedural strategies, 5) patient selection criteria, and 6) quality indicators for PCI.

Indications and patient selection criteria for PCI

Indications for PCI in CAD

PCI is indicated for various clinical scenarios in the management of CAD. It is performed in stable CAD patients when medical therapy fails or if there is a significant disease affecting their quality of life [6,7]. Timely or expeditious PCI is strongly advised for patients presenting with unstable angina to prevent MI. In acute coronary syndrome (ACS), PCI is the favored approach for reperfusion in patients of ST-segment elevation MI (STEMI) and is indicated in high-risk patients with non-ST-segment elevation MI [1,8,9]. PCI is also indicated in the left main disease and multivessel disease, depending on lesion complexity and patient risk [8]. For chronic total occlusions (CTOs), PCI is contemplated for patients experiencing symptoms who also exhibit evidence of myocardial ischemia and viable myocardium [10]. Furthermore, in cases of stenosis occurring after coronary artery bypass grafting (CABG), PCI may be employed as a means to reinstate proper blood flow [11]. Ongoing research explores emerging indications for PCI, including left ventricular dysfunction, microvascular angina, CTOs with symptoms, and ischemia-driven revascularization in high-risk scenarios [12]. Overall, PCI assumes a pivotal role in the comprehensive management of CAD, addressing diverse clinical presentations and improving patient outcomes.

Patient Selection Factors for PCI

Patient selection for PCI involves a comprehensive evaluation of clinical characteristics, coronary anatomy, and procedural risk. The decision to proceed with PCI is contingent upon a comprehensive evaluation of the patient's symptomatic status, hemodynamic stability, comorbidities, and risk stratification. Symptomatic patients with significant CAD that affect their quality of life are considered for PCI [13]. Comorbidities, including heart failure, renal impairment, and advanced age, are evaluated to assess procedural risk and guide treatment decisions [14,15]. The anatomical complexity of coronary lesions, including factors like severe calcification or bifurcation disease, along with the presence of left main or multivessel disease influences the need for revascularization and choice between PCI and CABG [16]. Procedural risk assessment incorporates patient factors and procedural considerations to estimate the risk-benefit ratio [17]. Risk scores, such as the EuroSCORE and SYNTAX Score, aid in assessing procedural risk and predicting outcomes following PCI [10,18].

Comparison of coronary artery revascularization guidelines

ACS represents a set of disorders marked by diminished supply to the myocardium. Both guidelines, 2021 ACC/AHA/SCAI and 2018 ESC, agree that for STEMI patients, primary PCI is the optimal revascularization approach if performed within 12 hours after the onset of symptoms or even later if evidence of persistent ischemia exists. However, there are differences between the two guidelines regarding the timing and extent of revascularization, the choice and duration of antithrombotic therapy, and IVUS use. The ACC/AHA/SCAI guideline suggests that complete revascularization should occur during primary PCI or within 45 days after STEMI in hemodynamically stable patients with multivessel disease. On the other hand, the ESC guideline recommends that complete revascularization should be carried out during primary PCI for only selected patients with cardiogenic shock or ongoing ischemia and suggests considering staged revascularization within 72 hours after STEMI in patients with hemodynamically stable disease [19,20].

Another example is that ACC/AHA/SCAI and ESC guidelines recommend at least 12-month dual antiplatelet therapy (DAPT) following PCI post-ACS. However, the ACC/AHA/SCAI suggests using ticagrelor or prasugrel over clopidogrel in most cases due to their greater efficacy. The ESC guideline recommends using ticagrelor over clopidogrel only in high-risk patients with diabetes mellitus or recurrent ischemic events. It offers an option to switch ticagrelor/prasugrel to clopidogrel within 12 months after PCI if the patient experiences trouble tolerating the medication or has a high-bleeding risk [19,20].

One additional example pertains to IVI during PCI, with both guidelines suggesting its use to optimize stent implantation, mainly through OCT or IVUS. However, the ACC/AHA/SCAI guideline recommends sonography-guided PCI more strongly than tomography-guided PCI based on more robust evidence from randomized trials showing improved outcomes with sonography-guided revascularization compared to angiography-guided revascularization. The ESC guideline does not differentiate between IVUS and OCT and states that both techniques can be used interchangeably depending on availability and operator preference [19,20].

Recent advancements in PCI techniques

Introduction to DES: Understanding DES, Their Mechanism of Action, and Their Comparison With Bare-Metal Stents (BMS)

One of the major advancements in PCI devices is the emergence of DES, tiny coils of metallic mesh placed into constricted or obstructed coronary or peripheral arteries, maintaining their patency to improve blood flow. Unlike BMS, DES have a medication-coated surface that is delivered gradually, preventing blood clot and subsequent scar tissue formation, which could again lead to artery narrowing (a condition called restenosis) [21]. DES obtained regulatory approval from European and American authorities in the years 2002-2003. Since then, numerous clinical trials have shown their effectiveness in reducing restenosis rates compared to BMS, irrespective of the type of lesion or clinical syndrome [22]. The drugs used in DES include paclitaxel, sirolimus, everolimus, and zotarolimus, among others [23].

Evolution of DES Generations: First-Generation DES (G1-DES), Second-Generation DES (G2-DES), and Introduction to Biodegradable Polymers

The evolution of DES occurred across several generations. The initial iteration of DES involved a stainless-steel framework coated with either sirolimus or paclitaxel, evaluated in trials such as RAVEL, SIRIUS, and TAXUS [24]. G2-DES, which contained everolimus or zotarolimus, were more effective in tests and featured a cobalt-chromium framework with various polymer coatings that enabled thinner struts, better flexibility, deliverability, more excellent biocompatibility, improved pharmacokinetic profiles, and enhanced endothelial regeneration. The DES of the second generation are currently the most widely used and were assessed in the trials of ENDEAVOUR and SPIRIT [25-27]. Clinical trials are now being conducted to evaluate the efficacy of third-generation DES, which features biodegradable polymers or fully bioresorbable vascular scaffolds [28,29].

Benefits and Effectiveness of DES and Impact on Target Vessel Revascularization, MI, and Mortality

Studies have shown that DES demonstrate a substantial reduction in the likelihood of restenosis compared to their counterparts. DES usage was also related to a reduced likelihood of MI and associated mortality in more extensive observational studies conducted in real-world settings without randomization, with an acceptable chance of selection bias and residual confounding. Target vessel revascularization is significantly reduced when DES are used as compared to BMS, as shown in both observational studies as well as randomized controlled trials (RCTs). Kirtane et al. suggested that DES have a good safety profile and effectiveness, both on and off-label. However, disparities exist between the findings of RCTs and observational studies when comparing the effectiveness data of the two rival stent types [30]. Another study by Ananthakrishna et al. suggested that the overall benefit of DES is primarily attributed to a reduced target lesion revascularization, but without a significant impact on overall mortality rates [31]. In a meta-analysis of eight RCTs performed by Changal et al., G2-DES were found to significantly decrease the risk of target lesion revascularization, heart attack, and mortality from all causes, compared to BMS. However, the disparity in overall mortality rates was not found with the use of G1-DES [32].

Adequate prolonged post-treatment surveillance information is lacking for precise assessment of stent thrombosis rates among patients receiving coronary stenting for large cohorts. According to a comparative study by Tada et al., the incidence of stent thrombosis remained significantly greater with both generations of DES for up to one year, when compared to BMS. However, after one year, stent thrombosis risk remained considerably greater solely in relation to G1-DES as compared to BMS [33].

It is important to note that there may be variations in the stent types used in studies, the patient's characteristics, and the follow-up duration, which could affect the results. Additionally, studies evaluated different endpoints, which makes direct comparisons challenging. Despite some studies demonstrating a correlation between DES and better outcomes than BMS, the evidence is inconclusive. Further research is necessary to determine the optimal stent type for coronary artery interventions.

Bioresorbable Vascular Scaffolds as an Advancement and Comparative Trials With DES

DES have been the standard-of-care for PCI for many years. However, recent advancements in DES technology have improved their efficacy and safety profile. One example is the development of BVS, which dissolve over time, eliminating the need for permanent metal scaffolds in the arteries [34].

In recent years, there has been notable interest within the medical community regarding the use of BVS compared to DES in various RCTs for treating CAD [35]. After one year of monitoring, in the ABSORB III clinical trial, Stone et al. assessed how effective BVS are, compared to everolimus-eluting stents (EES) and found that BVS was non-inferior to EES in regard to mortality from all causes of death from cardiovascular disorders including MI, and failure of the target lesion (ischemia-induced revascularization of the target lesion and target lesion-related MI) [36]. However, Kereiakes et al. reported increased rates of adverse events, mainly target lesion-related MI and scaffold thrombosis, between one and three years which continued to accumulate up to three years, with the use of BVS [37]. In the same study, Kereiakes et al. found cumulative adverse event rates at the five-year follow-up to be higher following the use of BVS compared to EES. Similarly, cumulative five-year adverse event rates following BVS were higher than with EES. The time of elevated risk for BVS, on the other hand, stopped after three years, coinciding with total scaffold resorption [38].

In the EverBio-2 trial, Puricel et al. evaluated the roles of different immunosuppressant medication coatings (everolimus and biolimus) in DES (everolimus and biolimus) against everolimus-eluting BVS and found that at the nine-month follow-up, these new-generation DES (everolimus and biolimus) were not better than BVS as far as angiographic late lumen loss is concerned, among other clinical outcomes [39]. After a five-year follow-up, Schukraft et al. revealed comparable angiographic and clinical outcomes between patients treated with BVS and DES. Moreover, within a selected subgroup, the angiographic results were found to be similar as well. Nonetheless, the trial was not adequately powered to draw definitive conclusions regarding clinical and angiographic endpoints [40].

Advances in Polymer Coatings and Risk vs. Benefit Analysis

The use of durable polymers in G1-DES has been suggested by Muramatsu et al. to potentially promote stent thrombosis and inflammation. Coatings of biodegradable polymers, derived from lactate or glycolate, increase the distribution of drugs to the vascular wall. These polymer coatings are capable of being fully resorbed through hydrolysis after drug release, with no prolonged effects [41]. Although using such polymers in subsequent generations of DES systems holds promise, many obstacles must be addressed before widespread clinical implementation. Biodegradable polymers are less likely to cause late thrombosis compared to the G1-DES. However, they may not offer the same advantage over the latest generations of such polymerized stent systems, for instance, durable polymer systems. Nonetheless, RCTs have not determined whether biodegradable polymer stents are superior to durable polymer systems and vice versa. RCTs have also not determined whether biodegradable polymer stents require shorter DAPT than durable polymer stents [42,43]. More trials, on a large scale, are needed to elucidate this research gap.

Ultrathin DES: The Impact of Stent Strut Thickness, Advantages of Ultrathin Infrastructure, and Reduced Risk of Stent Thrombosis

One recommended option is the utilization of G2-DES. Along with the antiproliferative drug and polymer used, further development in the DES metallic backbone, specifically exploring the impact of stent strut thickness on DES performance, holds promise. Recently, another potential advancement is using ultrathin DES, which have thinner struts (≤70 μm) and increased flexibility, improving their deliverability and reducing the risk of stent thrombosis [29,44].

Imaging modalities for PCI

Introduction to Intravascular Imaging Techniques

IVI techniques, including the use of IVUS and OCT, have achieved substantial improvements regarding PCI techniques [45]. Such imaging techniques give thorough information on coronary lesions, enabling more accurate stent optimization following DES insertion and pinpointing the origin of PCI complications.

Comparative Roles of IVUS and OCT: Grayscale Imaging, Tissue Identification Technology, and Applications in Lesion Assessment and Stent Optimization

IVUS generates grayscale, cross-sectional pictures of the artery wall and is currently refined with improved tissue identification technology [46]. It is most commonly used in cardiac interventional procedures to describe the lesion shape, measure atherosclerosis load, guide stent size, evaluate stent expansion, provide information on stent deployment accuracy, and identify procedural complications [47]. OCT, on the other hand, gives excellent-quality images of the coronary arteries, allowing for detailed assessments of stent placement and expansion. It also provides higher-resolution images, allowing for better stent apposition and tissue coverage visualization. These modalities improve procedural outcomes and reduce the risk of complications [48]. OCT provides higher imaging resolution, quicker pullback, angiography co-registration, various automated measures, and an easy-to-use interface. There is emerging evidence that OCT-guided PCI improves imaging and clinical outcomes, although there is still a shortage of data from randomized clinical studies. The cost and unpredictability of reimbursement are the primary barriers to increased acceptance of OCT guidance in PCI, with Japan at the top of the list [48].

The potential of pharmacological agents in preventing complications post-PCI

Pharmacological agents like antiplatelet and anticoagulant therapies are pivotal in avoiding stent thrombotic complications as well as minimizing the likelihood of ischemia-related events after PCI.

Dual Antiplatelet Therapy: Rationale, De-Escalation Strategies, and Duration Considerations

One such treatment regimen is DAPT, which involves the combination of two antiplatelet agents, typically aspirin and a P2Y12 receptor blocker, to lower the likelihood of thrombotic events in individuals who have a diagnosis of ACS or who have had a PCI [49]. In recent years, increasing interest has been in de-escalating DAPT to lower the bleeding risk complications while maintaining efficacy [50]. An active area of research is to find the ideal duration and combination of DAPT regimens. Several studies have been conducted to evaluate different regimens and their efficacy and safety profiles [51,52]. A meta-analysis by Tsigkas et al., comparing the use of a concise (less than three months) duration of DAPT to a longer duration (more than three months) in patients undergoing PCI with DES, was conducted. They found that very short DAPT substantially reduced the likelihood of massive bleeding and adverse clinical events without an increase in ischemia-related events. Consequently, the use of very short DAPT after PCI with DES was deemed tolerable and feasible [52].

Advancements in Antiplatelet Agents and Role in Personalized Medicine and Pharmacogenomics

Advancements in DAPT have also resulted in the emergence of newer, more powerful antiplatelet drugs, such as prasugrel and ticagrelor. These drugs have demonstrated better efficacy than clopidogrel in reducing the risk of ischemia-related events following PCI [53]. The duration of DAPT may vary depending on patient characteristics, the type of stent utilized, and the likelihood of thrombotic events vs bleeding complications. For individuals at high risk of bleeding, for example, a shorter period of DAPT may be explored. However, a more substantial duration may be suggested in individuals at high risk of thrombotic events [54].

Another area of advancement in DAPT is personalized medicine, which involves tailoring antiplatelet therapy to an individual's genetic makeup to optimize treatment outcomes and minimize bleeding risk. This approach is known as pharmacogenomics and involves testing for genetic variations that affect how an individual's body metabolizes and responds to antiplatelet medications. Notarangelo et al., suggested that incorporating genetic data related to clopidogrel metabolism, along with considering patients' clinical characteristics, can significantly reduce the incidence of both ischemic and bleeding events compared to standard practice [55,56]. 

Procedural strategies for improved outcomes

Radial Artery Access: Advantages, Limitations, Reduced Complications, and Patient Selection

Procedural strategies, such as radial artery access and complete revascularization, have also improved patient outcomes after PCI. While, historically, interventional cardiologists have commonly utilized the femoral artery as the access point for PCI, the radial artery has gained popularity recently due to its several advantages. In contrast to femoral artery access, radial artery access offers a notable advantage of lower incidence of bleeding and vascular complications. This benefit is because the radial artery is close to the skin surface, making it easier to compress and minimize bleeding [57]. Additionally, patients can sit up and move around sooner after the procedure, resulting in faster recovery times and shorter hospital stays [58]. Radial artery access may be the preferred choice for individuals with obesity, peripheral vascular disease, or prior femoral access complications [59]. However, there are also some limitations. One major limitation is that it may be technically challenging in patients with minor or tortuous radial arteries, which can result in procedure failure or a need to switch to a femoral approach. Additionally, radial artery access may not be feasible in patients with radial artery occlusion or spasm, which can occur in up to 10-20% of cases [57]. Another area for improvement is the need for specialized equipment and training for radial artery access, which may limit the widespread adoption of the technique [60].

Complete Revascularization and Beneficial Outcomes

Another significant advancement in PCI techniques is the concept of complete revascularization. Complete revascularization refers to restoring blood flow to all blocked arteries in the heart instead of just the ones causing symptoms. Studies have shown that complete revascularization improves outcomes, including reduced rates of major cardiovascular events and improved long-term survival [61,62].

Patient selection criteria for PCI

Patient selection criteria for PCI have been refined in recent years, focusing on appropriate use and minimizing the risk of complications. Established appropriate use criteria are available for determining the optimal selection of patients for coronary revascularization, considering factors such as the CAD severity and extent, symptomatic disease, and the expected therapeutic benefits [63]. The employment of the SYNTAX score plays a pivotal role in evaluating the intricacy associated with CAD and helps identify appropriate individuals for therapy and make treatment decisions [64].

Quality indicators for PCI care

Quality indicators are critical in assessing and enhancing the quality of care provided to patients undergoing PCI. The Canadian Cardiovascular Society has identified annual PCI volume as an essential quality indicator for PCI care [65]. Other critical processes of care indicators for PCI include aspirin prior to PCI, renal function, and urgent readmissions to acute care facilities [66]. The American College of Cardiology/American Heart Association Task Force on Performance Measures has established specific measures that evaluate crucial aspects of care for patients hospitalized with STEMI, including those undergoing PCI [67]. Additionally, for patients undergoing primary PCI, the first medical contact-to-first device time has emerged as a more comprehensive quality measure than the traditional door-to-balloon time [68].

Continuous monitoring of quality indicators

Continuous monitoring of quality indicators is crucial to ensure efficient and effective care delivery. Quality indicators should be set based on scientific concepts, experiences, literature searches, and discussions with experts within and outside the institution. Moreover, the numerator and denominator of the quality indicators should be strictly defined to ensure accuracy in the monitoring process. Trend analysis of the quality indicators can help identify areas where the quality of care may need to be improved [69].

## Conclusions

In conclusion, PCI remains a highly effective intervention for CAD, providing symptom improvement, reducing angina, and enhancing patient outcomes. The advent of DES has significantly decreased restenosis rates and the necessity for repeat revascularization procedures compared to BMS. To prevent stent thrombotic and ischemia-related complications post-PCI, antiplatelet and anticoagulant therapies are crucial. DAPT remains the established regimen to reduce thrombotic events during PCI with DES, and strategies for de-escalation can help balance effectiveness with the risk of bleeding. The use of very short DAPT after PCI with DES demonstrates tolerability and feasibility as compared to BMS. Recent advancements in DAPT, including prasugrel and ticagrelor, have demonstrated superior efficacy in preventing ischemic events following PCI compared to clopidogrel. Personalized medicine, through pharmacogenomic testing, allows for the optimization of antiplatelet therapy while minimizing bleeding risk. Procedural strategies such as radial artery access and complete revascularization have shown improved patient outcomes in PCI procedures. Radial artery access offers advantages such as reduced bleeding and vascular complications, faster recovery times, and shorter hospital stays. Complete revascularization, which ensures blood flow restoration to all blocked arteries, has been linked to better long-term survival and a lower occurrence of major cardiovascular events.

Patient selection criteria, including appropriate use criteria and the utilization of the SYNTAX score, assist in identifying individuals who will benefit from coronary revascularization based on disease severity, symptoms, and expected therapeutic outcomes. Continuous monitoring of quality indicators is necessary to ensure the efficient and effective delivery of care and to identify areas where improvements can be made. In the future, further research is warranted to refine pharmacological regimens, explore innovative procedural strategies, and continuously improve the assessment of quality indicators.
